# 

**Published:** 1850-05

**Authors:** 


### CONTENTS

OF THE

### MEDICAL EXAMINER.

NEW SERIES—NO. LXV.—MAY, 1850.

### ORIGINAL COMMUNICATIONS.

Hydrocele of the Neck. By Thomas D. Mütter, M. D., Professor of Surgery in Jefferson Medical College. With cases and illustrations. (Clinic of Jefferson Medical College,) - - - 257
Case of Purulent Infection. Service of M. Roux. Hotel Dieu. Reported by J. H. Weir, M. D., Philadelphia, - - - 275

### BIBLIOGRAPHICAL NOTICES.

A Treatise on the Etiology, Pathology and Treatment of Congenital Dislocations of the Head of the Femur. Illustrated with plates. By John Murray Carnachan, M. D., Lecturer on Operative Surgery with Surgical and Pathological Anatomy, &c., 277
The Diseases of Females; including those of Pregnancy and Childbed. By Fleetwood Churchill, M.D., Author of the Theory and Practice of Midwifery and Diseases of Infants. A new American edition revised by the Author. With the notes of R. M. Huston, M. D., Professor of Materia Medica and General Therapeutics in Jefferson Medical College, &c., - - - - 287

Dietetical and Medical Hydrology. A Treatise on Baths; including Cold, Sea, Warm, Hot, Vapor, Gas, and Mud Baths; also, on the Watery Regimen, Hydropathy, and Pulmonary Inhalation: with a description of Bathing in Ancient and Modern Times. By John Bell, M. D., Member of the American Medical Association, &c. &c., - - - - - - - - - - 289

Surgical Anatomy. By John Maclise, Surgeon, - - - - 292

The Encyclopedia of Chemistry, Practical and Theoretical; embracing its applications to the Arts, Metallurgy, Mineralogy, Geology and Pharmacy. By James C. Booth, A. M., M. A. P. S., &c. Assisted by Campbell Morfit, - - - - - - - 293

The Medical Practitioners' and Students' Library, No. 5. The American Medical Formulary: based upon the United States and British Pharmacopœias. Including also numerous standard formulæ, derived from American and European authorities. Together with the medical properties and uses of Medicines; Poisons, their antidotes, tests, &c.; designed for the medical and pharmaceutical student. By John J. Reese, M. D., Lecturer on Materia Medica and Therapeutics in the Philadelphia Medical Institute, &c., - - - - - - - - 295

The Druggists' General Receipt Book: containing numerous recipes for patent and proprietary medicines, Druggists' nostrums, etc.; Factitious Mineral Waters, and powders for preparing them; with a Veterinary Formulary, and table of veterinary Materia Medica; also recipes for perfumery and cosmetics; beverages, dietetic articles and condiments; trade chemicals, miscellaneous compounds used in the arts, domestic economy, &c.; with useful tables and memoranda. By Henry Beasley, - - - - - - - - - - 295

### EDITORIAL.

Facts vs. Figures, - - - - - - - - - - 298

Assimilated Rank in the Navy, - - - - - - - - 302

Professorial Changes and Appointments, - - - - - - 309

Hampden Sidney College, Richmond, Va., - - - - - - 310
Medical Graduates and Students, - - - - - - - - - 310
St. Louis Medical and Surgical Journal, - - - - - - 310
Pennsylvania State Medical Society, - - - - - - - 310
The alleged Employment of Chloroform by Thieves. By John Snow, M. D., - - - - - - - - - - - - - - 316
Deaths in Philadelphia from March 23d to April 20th, 1850. Reported by Mr. James Aitken Meigs, M. D., Student of Medicine, - - - - - - - - - - - - - - - - 317
Removal of Foreign Bodies from the Ear Passages, - . . - 318

